# Prevalence and risk factors of burden among caregivers of older emergency department patients

**DOI:** 10.1038/s41598-023-31750-1

**Published:** 2023-05-04

**Authors:** Tessel Zaalberg, Dennis G. Barten, Caroline M. van Heugten, Petra Klijnsma, Lieve Knarren, Ytje Hiemstra, Roel A. J. Kurvers, Anita W. Lekx, Simon P. Mooijaart, Maryska Janssen-Heijnen

**Affiliations:** 1 Department of Emergency Medicine, VieCuri Medical Center, P.O. Box 1926, 5900 BX Venlo, The Netherlands; 2 Department of Neuropsychology and Psychopharmacology, Maastricht University, Maastricht, The Netherlands; 3 Limburg Brain Injury Center, Maastricht, the Netherlands; 4 School for Mental Health and Neuroscience, Maastricht University Medical Center, Maastricht, The Netherlands; 5 Department of Geriatric Medicine, VieCuri Medical Center, Venlo, The Netherlands; 6 Department of Internal Medicine, VieCuri Medical Center, Venlo, The Netherlands; 7 Caregiver Representative, MantelzorgNL, Zeist, The Netherlands; 8 Department of Pediatrics, Maastricht University Medical Center, Maastricht, The Netherlands; 9 Sleep Medicine Center Kempenhaeghe, Heeze, The Netherlands; 10 Department of Gerontology and Geriatrics, Leiden University Medical Center, Leiden, The Netherlands; 11 Department of Clinical Epidemiology, VieCuri Medical Center, Venlo, The Netherlands; 12 Department of Epidemiology, Faculty of Health, Medicine and Life Sciences, GROW School for Oncology and Reproduction, Maastricht University, Maastricht, The Netherlands

**Keywords:** Health care, Medical research, Risk factors

## Abstract

The number of older individuals that live independently at home is rising. These older individuals often rely on caregivers who have a similar age and health status. Therefore, caregivers may experience a high burden. We determined the prevalence and associating factors of burden among caregivers of older patients in the emergency department (ED). A cross-sectional study of primary caregivers of patients aged ≥ 70 years visiting the ED of a Dutch teaching hospital was performed. Structured interviews were conducted with patients and their caregivers. Caregiver burden was measured using the caregiver strain index (CSI). Additionally, data from questionnaires and medical records were extracted to determine potential associating factors. Univariate and multivariate regression analyses were conducted to identify independent determinants for burden. Seventy-eight caregivers (39%) experienced a high burden. Multivariate analysis showed a significant association between high caregiver burden and patients with cognitive impairment or dependency for instrumental activities of daily living (IADL) and more self-reported hours of care per day. Almost 40% of older patients in the ED have a caregiver who experiences a high burden. Formal assessment in the ED may help provide adequate care to the patients and their caregivers.

## Introduction

In many developed countries, the number of older individuals who live independently at home is increasing^1^. In 2011, almost half of all older adults in the US reported receiving help with daily activities. Of these, 83% lived in community settings and nearly 1 in 5 received help with their most basic self-care or mobility activities^2^. A recent study in the Netherlands showed that 95% of older ED patients lived independently at home prior to their visit. A caregiver was reported by 51% of patients^3^. Overall, 16% of caregivers of patients with cancer experience a high to severe burden^4^, and for caregivers of stroke survivors and patients with dementia this burden is even higher, approaching 35% and 60%, respectively^5,6^. Furthermore, the number of hours of care delivered is associated with the occurrence of burnout among caregivers^7,8^. Caregivers with a high burden may cause potential health risks for patients through inadequate care, medication mistakes, and elder abuse or neglect^9^.

Acutely presenting older patients in the emergency department (ED) often have caregivers with similar age^10,11^ and health status, which may result in a fragile balance regarding care needs at home. Surprisingly, information on the prevalence and risk factors of caregiver burden is limited. Especially little is known about their level of burden when older patients seek acute medical care. Any evidence linking burden of caregivers with acute care utilization will potentially enhance community services to avoid acute care.

In this cross-sectional study, we aimed to determine the prevalence and associating factors of caregiver burden of acutely presenting older patients in the ED.

## Methods

### Study design

We conducted a cross- sectional study in the ED of VieCuri Medical Center in Venlo, the Netherlands. This is a teaching hospital and a level 2 trauma center with an annual ED census of 25,000 patients. In 2019, 33% of the patients who presented to the ED were ≥ 70 years old. In the Netherlands, primary healthcare is well developed and accessible for patients 24 h a day. General practitioners (GPs) serve as gatekeepers to hospital care. During office hours, patients can consult their own GP, usually obtaining an appointment that day. Afterhours primary care is provided through GPs cooperatives. The majority of ED patients are referred by their GPs or by ambulance. Self-referrals compromise a small minority^12^.

This study was approved by the Institutional Review Board of VieCuri Medical Center, Venlo, the Netherlands (#458).

### Participant selection

Participants were recruited between November 6, 2019 and January 19, 2020, with an interruption of one week from December 25, to January 1. All patients ≥ 70 years of age were screened for eligibility by the ED’s treating physician. Their baseline characteristics were collected. If patients reported a caregiver, both the patients and the caregiver were included for further analysis. A caregiver was defined according to Kent et al. as “individuals who provide care that is typically uncompensated and usually at home, which involves significant amounts of time and energy for months or years, and which requires the performance of tasks that may be physically, emotionally, socially or financially demanding.”^13^ Exclusion criteria for patients were living in a nursing home, unwilling to participate, unable to participate due to language barriers, highest triage category (red) using the Manchester triage system^14^, and inability to provide informed consent (with no legal representative available). Patients and their caregivers were provided written information about the study and the treating physician obtained informed consent.

### Data collection

A structured medical record review (appendix 1) of patients was performed in which information was collected to identify potential risk factors for caregiver burden. Patients and caregivers were interviewed separately by one researcher (T.Z.) using questionnaires. The interview took place during the ED visit or within 7 days after the ED visit by telephone.

The following information about patients was collected from questionnaires: age; sex; marital status; highest level of education (divided into two categories: low [unfinished, primary, secondary] and high education [vocational and tertiary studies]); employment; nationality; reason for ED visit (trauma-related or not); mode of ED referral; cognitive impairment (defined as official diagnosis of dementia determined by a geriatrician); active malignancy; homecare; number of caregivers; age adjusted Charlson Comorbidity Index (ACCI) presented as estimated 10-years survival^15^. For the ACCI we looked into the medical record of the patient; Acutely Presenting Older Patients (APOP) score (risk of functional decline or mortality in three months)^16^; Activities of Daily Living (ADL) KATZ score^17^ and Instrumental activities of daily living (IADL) score^18^. The ADL-KATZ score ranges from 0 to 6 and a higher score corresponds with higher dependency. The IADL score measures, seven everyday functional competence through self-reporting. Each item could score 2 points. Score ranges from 0 to 8 (dependent) or 9–14 (independent).

The caregiver information collected included: age; sex; nationality; relationship to the patient; highest level of education (divided into two categories: low [unfinished, primary, secondary] and high education [vocational and tertiary studies]); employment; self- reported number of hours of caring for the patient; distance to the patient; term of care provided; clinical frailty scale (CFS)^19^ and caregiver strain index (CSI)^20^.

The CSI is a brief and easy 13-question tool used to quickly identify potential burden of caregivers that contains subjective and objective elements. Positive responses to ≥ 7 questions indicate a high level of strain. CSI was considered low when scored 1–3 and intermediate when scored 4–6. There is a minimum of one question for each of the following domains: employment, financial, physical, social and time^20^. Caregivers with a CSI index ≥ 7 completed this questionnaire again four weeks after the initial ED visit to determine whether the burden persisted.

### Sample size

We aimed to include 200 caregivers based on previous research with a comparable study setting^3,21^. With an expected burden of 20%, we estimated including 160 caregivers without and 40 caregivers with burden.

### Statistical analysis

SPSS version 24 (International Business Machines Cooperation, Amsterdam, the Netherlands) was used for statistical analyses. Baseline characteristics were presented as numbers and percentages for categorical variables and means or medians for continuous variables. Pearson’s chi squared t- test and the Mann Whitney u- test were used to determine the association between experienced burden (yes/no) and dichotomous and categorical patient and caregiver characteristics. Univariate logistic regression analyses were conducted to analyze the association between patient and caregiver characteristics as independent variables, and the caregiver burden as dependent variable. A high burden was defined as a CSI > 7.^22^ Characteristics that were associated with burden in univariate analyses were included in a multivariate logistic regression analysis to investigate which characteristics were independently associated with burden. Age and sex of both patient and caregiver were also included in this multivariate regression analysis. We calculated odds ratios (ORs) for burden with 95% confidence intervals (CI) and a *p* value of < 0.05 was considered significant.

### Ethics approval and consent to participate

This study was approved by the Institutional Review Board of VieCuri Medical Center, Venlo, the Netherlands (#458). Patients and their caregivers were provided written information about the study and the treating physician obtained written informed consent. All methods were performed in accordance with the relevant guidelines and regulations.

## Results

### Patients

**Figure 1:**
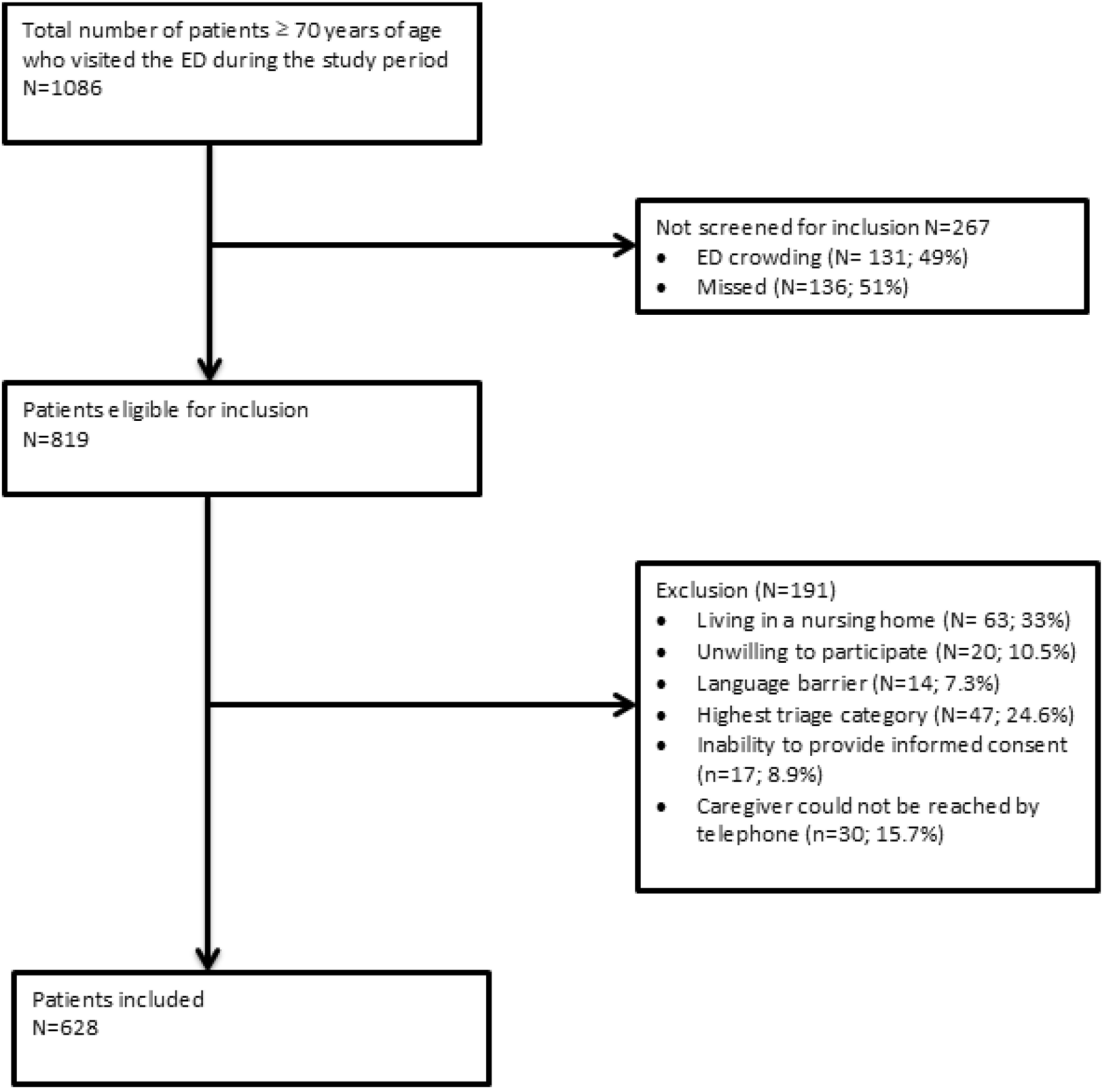
Patient inclusion and exclusion flow chart.

During the study period, 1,086 patients aged ≥ 70 years visited the ED. Of these, 267 were not screened for inclusion (Fig. 1). Of the remaining 819 patients, 628 were included in the analyses. The reasons for exclusion (n = 191) were: living in a nursing home (n = 63; 33%), unwilling to participate (n = 20; 10.5%), language barrier (n = 14; 7.3%), highest triage category (n = 47; n = 24.6%), inability to provide informed consent (n = 17; 8.9%), or caregiver could not be reached by telephone (n = 30; 15.7%) (Fig. 1). A primary caregiver was reported by 31.8% of the 628 study participants (n = 200).

Compared to patients without a caregiver, patients with a caregiver were on average older (78 vs. 82 years). The other baseline characteristics were similar (Table 1).

**Table 1:** Baseline characteristics.

		Patients without a caregiver n = 428	Patients with a caregiver n = 200
Age in years, mean (SD)		78 (6.454)	82 (6.692)
Gender (%)	Male	223 (52.1)	99 (49.5)
	Female	205 (47.9)	101 (50.5)
Arrival (%)	By ambulance	76 (17.7)	50 (25)
	Referred by GP		
	By ambulance	58 (13.6)	52 (26)
	Own transport	225 (52.6)	81 (40.5)
	Other	69 (16.1)	17 (8.5)
Specialty (%)	Internal medicine	78 (18.2)	50 (25)
	Traumatology	141 (33)	49 (24.5)
	Surgery	49 (11.4)	21 (10.5)
	Neurology	56 (13.1)	31 (15.5)
	Pulmonary medicine	50 (11.7)	40 (20)
	Other	54 (12.6)	9 (4.5)
Presenting time (%)	Office hours (8.00–17.00)	256 (59.8)	132 (66)
	Evening hours (17.01–0.00)	57 (13.3)	24 (12.0)
	Night hours (0.01–7.59)	29 (6.8)	11 (5.5)
	Weekend hours	86 (20.1)	33 (16.5)

*SD* Standard deviation, *GP* General practitioner.

### Patients and their caregivers

Of the patients with a caregiver, 20% had an active malignancy and 22.5% were cognitively impaired. The percentage of patients living alone was 42.5%. The mean age (SD) of the caregivers was 66 (12.4) years, 67.5% were female and 51% of the caregivers were the spouse or partner of the patient. Among the 200 caregivers in this study, 78 (39%) experienced a high burden with a CSI score ≥ 7. Both the patient age and caregiver age were not significantly associated with burden.

Compared to caregivers with a low burden, caregivers with a high burden were more often caregivers of a patient with cognitive impairment (43.6% vs. 9%; *p *= 0.000), a higher ADL score (20.5% vs. 6.6%; *p *= 0.003) and a lower IADL score (93.6% vs. 63.9%; *p *= 0.000). A higher ADL score is associated with more dependence, while a higher IADL score is associated with more independence. The median number of self-reported hours of care per day by caregivers was significantly higher among caregivers with a high burden, both for partners in the caregiver role (24.00 h (5.30–24.00) vs. 2.00 h (1.00–4.00); *p *= 0.000) and children (2.00 h (1.00–4.37) vs. 1.00 h (0.34–1.50); *p *= 0.000) as caregiver.

Patient and caregiver characteristics are summarized in Table 2.

**Table 2:** Associations of caregiver burden with patient and caregiver characteristics.

**Patient Characteristics**		Burden + n = 78	Burden – n = 122	*p*- value
Age in years, mean (SD)		82.2 (6.5)	82.5 (6.9)	0.717
Gender (%)	Male Female	40 (51.3) 38 (48.7)	59 (48.4) 63 (51.6)	0.687
Living alone (%)		29 (37.2)	56 (45.9)	0.224
Active malignancy (%)		13 (16.7)	27 (22.1)	0.346
Official diagnosis cognitive impairment* (%)		34 (43.6)	11 (9)	0.000
Receiving homecare (%)		44 (56.4)	56 (45.9)	0.147
Estimated 10 years survival (%)	0–50% > 50%	69 (88.5) 9 (11.5)	98 (80.3) 24 (19.7)	0.131
APOP score (%)	Low risk of adverse outcome High risk of adverse outcome	31 (39.7) 47 (60.3)	64 (52.5) 58 (47.5)	0.079
ADL KATZ (%)	Low level of dependency High level of dependency	62 (79.5) 16 (20.5)	114 (93.4) 8 (6.6)	0.003
IADL	Dependent Independent	73 (93.6) 5 (6.4)	78 (63.9) 44 (36.1)	0.000
Level of education (%)	Low (unfinished – secondary) High (vocational – tertiary)	57 (73.1) 21 (26.9)	84 (68.9) 38 (31.1)	0.523
Arrival	By ambulance Referred by GP By ambulance Own transport Other	22 (28.2) 22 (28.2) 24 (30.8) 10 (12.8)	28 (23.0) 30 (24.6) 57 (46.7) 7 (5.7)	0.087
Trauma-related ED visit (%)		24 (30.8)	25 (20.5)	0.099
Specialty (%)	Internal medicine Traumatology Neurology Surgery Pulmonary medicine Other	21 (26.9) 24 (30.8) 15 (19.2) 5 (6.4) 11 (14.1) 2 (2.6)	29 (23.8) 25 (20.5) 16 (13.1) 16 (13.1) 29 (23.8) 7 (5.7)	0.189
Presenting time (%)	Office hours (8.00–17.00) Evening hours (17.01–0.00) Night hours (0.01–7.59) Weekend hours	46 (59.0) 10 (12.8) 5 (6.4%) 17 (21.8%)	86 (70.5%) 14 (11.5%) 6 (4.9%) 16 (13.1%)	0.335
*Caregiver Characteristics*				
Age in years, mean (SD)		66.1 (11.6)	66.4 (13.0)	0.846
Gender (%)	Female Male	56 (71.8) 22 (28.2)	79 (64.8) 43 (35.2)	0.300
Employment (%)		22 (28.2)	48 (39.3)	0.107
Caregiver living with patient (%)		45 (58.4)	61 (50)	0.245
Level of education (%)	Low (Unfinished – secondary) High (vocational – tertiary)	30 (38.5) 48 (61.5)	48 (39.3) 74 (60.7)	0.901
Relationship with patient (%)	Partner Children Daughter Son Other	42 (53.8) 25 (32.1) 9 (11.5) 2 (2.6)	61 (50) 30 (24.6) 23 (18.9) 8 (6.5)	0.347
Self-reported number of hours of care/day, median (IQR)	Partner Children	24.0 (5.30–24.00) 2.0 (1.00–4.37)	2.0 (1.0–4.0) 1.0 (0.3–1.5)	0.000
Self-reported number of hours or care/day (%)	≤ 1 h 1–23 h 24 h	12 (15.4) 37 (47.4) 29 (37.2)	58 (47.5) 51 (41.8) 13 (10.7)	0.000
Distance to the patients km, median (IQR)	Partner Children	0.0 (0–0) 1.0 (0.28–5)	0.0 (0–0) 2.0 (1.0–5.5)	0.484 0.136
Distance to the patients km (%)	Living together ≤ 10 km > 10 km	45 (57.7) 29 (37.2) 4 (5.1)	61 (50) 50 (41) 11 (9)	0.435
Term of care provided years, median (IQR)	Partner	5.0 (2.4–15.0)	5.0 (1.5–11.5)	0.266
	Children	4.0 (2.0–10.0)	6 .0 (2.0–10.0)	0.371
CFS (%)	Very Fit Well Managing Well Vulnerable	8 (10.3) 30 (38.5) 29 (37.2) 14 (11.5)	21 (17.2) 49 (40.2) 38 (31.1) 11 (14.1)	0.493

Burden +  = Caregiver strain index ≥ 7. Burden – = Caregiver strain index < 7.

*ED* Emergency Department. *SD* Standard deviation; *GP* general practitioner;

*APOP* acutely Presenting Older Patients. An APOP score of 0–44 is defined as a low risk of adverse outcomes and an APOP score ≥ 45 is defined as a high risk of adverse outcomes.

*ADL KATZ score* Activities of Daily living. Dependency of 0–3 items is defined as a low level of dependency, and dependency of 4–6 items is defined as a high level of dependency.

*IADL score* Instrumental activities of daily living. Dependent: defined as an IADL score 0–8. Independent: defined as an IADL score > 8.

*IQR* Interquartile range. *CFS* clinical frailty scale.

*Diagnosed by a geriatrician (including dementia, Alzheimer’s disease, frontotemporal degeneration, Lewy body disease, traumatic brain injury (TBI) and neurocognitive issues due to Parkinson’s disease).

In the univariate logistic regression analyses, cognitive impairment (OR = 7.80), ADL KATZ score with a high level of dependency (OR = 3.68), IADL dependency (OR = 8.24) and self-reported number of hours of care per day (OR = 3.51 for 1–23 h vs < 1 h and OR = 10.78 for 24 h vs < 1 h) were significantly associated with a high caregiver burden (Table 3).

**Table 3:** Analyses.

		Univariate regression OR (95% CI)	Multivariate regressionOR (95% CI)
**Patient Characteristics**			
Age (per year)		1.01 (0.97–1.05)	1.01 (0.95–1.06)
Gender	Female Male	1 1.12 (0.64–1.99)	1 0.84 (0.40–1.78)
Living alone	No Yes	1 0.70 (0.39–1.25)	NI
Active malignancy	No Yes	1 0.70 (0.34–1.47)	NI
Official diagnosis cognitive impairment*	No Yes	1 7.80 (3.63–16.75)	1 3.90 (1.64–9.30)
Receiving homecare	No Yes	1 1.53 (0.86–2.702)	NI
Estimated 10 years survival	0–50% > 50%	1 0.53 (0.23–1.21)	NI
APOP score	Low risk of adverse outcome High risk of adverse outcome	0.60 (0.34–1.06) 1	NI
ADL KATZ	Low level of dependency High level of dependency	1 3.68 (1.49–9.08)	1 1.08 (0.36–3.29)
IADL	Independent Dependent	1 8.24 (3.10–21.91)	1 4.46 (1.50–13.23)
Level of education	Low (Unfinished – secondary) High (vocational – tertiary)	1 0.81 (0.43–1.53)	NI
Presenting time	Office hours (8.00–17.00) Evening hours (17.01–0.00) Night hours (0.01–7.59) Weekend hours	1 1.34 (0.55–3.24) 1.59 (0.45–5.38) 1.99 (0.92–4.29)	NI
**Caregiver Characteristics**			
Age		0.998 (0.98 -1.02)	0.97 (0.94–1.00)
Gender	Female Male	1 0.722 (0.39–1.34)	1 0.55 (0.24–1.22)
Employment	No Yes	1 0.61 (0.33–1.12)	NI
Caregiver living with patient	No Yes	0.711 (0.40–1.27) 1	NI
Level of education	Low (Unfinished – secondary) High (vocational – tertiary)	0.96 (0.54–1.73) 1	NI
Relationship with patient	Partner Children Other	1 0.93 (0.52–1.67) 0.36 (0.07–1.80)	NI
Self-reported number of hours of care/day	≤ 1 h 1–23 h 24 h	1 3.51 (1.65–7.44) 10.78 (4.37–26.58)	1 2.80 (1.22–6.44) 8.21 (2.53–26.62)
Distance to the patients (km)	Living together ≤ 10 km > 10 km	1 0.77 (0.43–1.43) 0.49 (0.15–1.65)	NI
Term of care provided (years)	Partner Children	1.01 (0.97–1.05) 0.97 (0.90–1.03)	NI
CFS	Very Fit Well Managing Well Vulnerable	1 1.61 (0.63–4.08) 2.00 (0.78–5.16) 2.06 (0.66–6.41)	NI

*APOP* Acutely Presenting Older Patients. An APOP score of 0–44 is defined as a low risk of adverse outcomes, and an APOP score ≥ 45 is defined as a high risk of adverse outcomes.

*ADL KATZ score* Activities of Daily living. Dependency of 0–3 items is defined as a low level of dependency, and a dependency of 4–6 items is defined as a high level of dependency.

*IADL score* Instrumental activities of daily living. Dependent is defined as an IADL score 0–8. Independent is defined as an IADL score > 8.

*Diagnosed by a geriatrician (including dementia, Alzheimer’s disease, frontotemporal degeneration, Lewy body disease, traumatic brain injury (TBI) and neurocognitive issues due to Parkinson’s disease).

*CFS* Clinical frailty scale, *NI* Not Included.

In the multivariate logistic regression analysis, cognitive impairment of the patient (OR = 3.91), IADL dependency (OR = 4.46), and self- reported number of hours of care per day by the caregiver (OR = 2.80 for 1–23 h vs < 1 h and OR = 8.21 for 24 h vs < 1 h) were independently associated with high caregiver burden (Table 3).

Four weeks after ED presentation, the 78 caregivers with a high burden were reassessed for possible persistence of burden. Six (7%) were lost to follow-up because they could not be reached by telephone. Of the 71 caregivers reached by telephone, the burden persisted in 49/71 individuals (69%).

## Discussion

This study to assess the prevalence and associating factors for caregiver burden of acutely presenting older patients in the ED found that approximately 40% of the caregivers experienced a high burden. These findings demonstrate that burden is highly prevalent among caregivers presenting at the ED. We identified an association between caregiver burden and patients with cognitive impairment or IADL dependency, as well as the number of self-reported hours of care.

Most prior studies on caregiver burden were conducted on specific patient populations such as older patients with dementia, cancer, or advanced illness^4,23–25^. In our cohort, caregiver burden was relatively high compared to other studies^4,5,26^. This could be explained by several patient and caregiver characteristics. First, our study included a relatively high number of dependent care recipients such as patients with cognitive impairment, stroke survivors and an active malignancy. However, this profile is probably representative for the community dwelling older patients in our society. Second, more than half of the caregivers were female. It has been shown that women more often care for ill relatives and are more likely than men to suffer from depression regarding caregiving tasks^27^. Third, the number of multigenerational caregivers, those who care for children and parents, is rising as life expectancies increase. Increasing workloads and caring for a relative generally negatively affect the daily lives of caregivers and may contribute to their burden^28^. Fourth, the ED visit may have been the climax of a long, debilitating journey of illness and disability, which may be reflected in a high caregiver burden. Finally, the identification of caregivers depends on the definition: caregivers with no burden and few care tasks may not consider themselves as caregiver, which may be associated with an overestimation of the percentage of burden^29^.

This study confirms previous research that found a univariate association between caregiver burden and cognitive impairment, ADL dependency, and IADL dependency of patients^5,27,30,31^. After adjusting for the other variables, we found that only cognitive impairment and IADL dependency were associated with a high caregiver burden. This could be explained by the ADL support provided by homecare professionals. Homecare assistance has been reported by 50% of the participants. IADL includes tasks that are not always resolved by homecare professionals, such as grocery shopping, cooking, and assisting with patient transfers.

Caregiver burden does not only harm the caregiver, but is also associated with potential patient health risks. It may result in inadequate care, medication mistakes, and elder abuse or neglect^9^. Although it is likely that caregiver burden is associated with higher ED utilization, this has not been investigated. However, studies on unplanned hospital readmission of older patients found an association between caregiver burden and the risk of readmission^21^. Furthermore, social problems and insufficient social support have been identified as major drivers of ED use^32^.

Surprisingly, the patients’ children reported more years of caregiving than the patients’ partners. Children may have considered themselves caregiver sooner than patients’ partners. One possible explanation could be the shift in roles that was perhaps unwanted. We all provide some form of ‘caregiving’ for our spouses and children, but often not for our parents. Despite this, there was no association between both the term of care provided and the relationship between the caregiver and patient..

Approximately 60% of the caregivers experienced no high burden. In fact, caregiving was also associated with positive aspects or a positive appraisal. The caregiver may experience an improved relationship with the patient, a higher self-efficacy, and a sense of personal growth^33^. This may contribute to a better quality of life for the caregiver^34–36^. However, caregivers are often untrained for this challenging role. They may not have access to caregiving information or support, and caregiving education programs are not widely implemented^37^.This study has several limitations. First, the CSI was measured during presentation at the ED or shortly thereafter. Unfortunately, we did not indicate whether the interview was conducted at the ED or by phone. The timing of the interview may have changed the perception of the conversation, which could have influenced the score. However, the burden persisted in 49 caregivers (69%) four weeks after ED presentation, so this influence was probably limited. Persistent burden was observed in caregivers of patients that were admitted and of patients that were discharged from the ED. Unfortunately, whether the burden persisted or not was not linked to the patients’ disposition in our database. Future studies should assess if ED admission or discharge impacts caregiver burden. Second, some caregivers may have been missed because not all caregivers may consider themselves as such. Caregivers who provide long-term or intensive care more often consider themselves caregiver, which may contribute to overestimation of burden. Various caregiver definitions circulate in the medical literature, further complicating the interpretation of our findings^38^. Third, caregivers were not interviewed about their quality of life or other caregiving experiences. There may possibly be differences in experienced burden influenced by whether the caregiver voluntarily choose to act as a caregiver or if they were expected into the role. Caregivers may also experience positive aspects of care or positive appraisal such as caregiver satisfaction, but these aspects were not investigated. Fourth, this was a single-center study, and demographics as well as caregiver burden may differ between regions or healthcare systems. Fifth, it was not assessed whether caregivers also had to care for other individuals, which may have contributed to their burden. The persistence of burden was only assessed in caregivers with a CSI ≥ 7. It is therefore unknown what the course of burden was in the caregivers with a lower burden. Sixth, the CSI contains subjective and objective elements; any psychological comorbidity of the caregiver might influence these subjective elements. Seventh, the impact of cognitive impairment on caregiver burden may have been underestimated. The definition of cognitive impairment was very strict. The majority of individuals with mild to moderate cognitive impairment may not have this diagnosis, but do have significant needs. Eighth, differences in health care systems are important and may impact the generalizability of our findings. Our primary care system is well established, however, a system with less well developed primary care may be associated with increased caregiver burden. Finally, 267 of 1,086 patients were not screened for eligibility. In most of the missed cases, the site researcher (T.Z.) was not present and the treating physician did not screen the study patients. Also, ED crowding occurred multiple times during the study period, occasionally hampering inclusion. To reduce selection bias, the researcher was scheduled in random shifts. This “true random sampling” method has been shown to represent the overall population in more than 95% of the samples and it has a low probability of selection bias^39^.

This study’s strengths include its prospective design, participant recruitment, relatively large sample size of 200 patients, use of a validated caregiver burden scale, inclusion of all older patients referred to the ED regardless of the reason for their ED visit, and the fact that caregivers and patients were interviewed separately.

Future studies should be multicenter and multinational to assess whether the high caregiver burden occurs in other regions or countries with different healthcare systems. Whether there is an association between high caregiver burden and (low-urgent) ED utilization or negative outcomes should also be ascertained. If an association is found, it should be assessed if the caregiver burden could be used as a screening tool to identify ED patients who are frail or have a high risk of experiencing negative outcomes. Predicting outcomes of older ED patients is essential for delivering adequate care, but can be challenging^40^. New predictors outside the scope of classical medical or frailty-based data may therefore be of extra value. Furthermore, 30-day mortality after ED visit is more associated with frailty than triage urgency^41^. As such, caregiver burden may be a marker of system frailty (as opposed to individual frailty).

## Conclusion

Among older patients presenting in the ED, about 32% reports to have a caregiver. Of those caregivers, almost 40% experience a high burden. Formal assessment in the ED may help provide adequate care to the patients and their caregivers, but more research is needed to establish whether caregiver burden is associated with frailty or negative outcomes in older ED patients.

## Supplementary Information

Supplementary Information.

## Data Availability

The datasets generated and analysed during the current study are available from the corresponding author on reasonable request.

## Abbreviations

ED: Emergency department
GPS: General practitioners
ACCI: Age adjusted charlson comorbidity index
APOP: Acutely presenting older patients
ADL: Activities of daily living
IADL: Instrumental activities of daily living
CFS: Clinical frailty scale
CSI: Caregiver strain index
ORs: Odds ratios
CI: Confidence intervals
