# Supplementary material for: Prevalence and risk factors of burden among caregivers of older emergency department patients
Source: Sci Rep. 2023 May 4;13:7250. doi: 10.1038/s41598-023-31750-1 (PMC10160020; doi:10.1038/s41598-023-31750-1)
Supplement: Supplementary file 1 — Supplementary Information. [file 41598_2023_31750_MOESM1_ESM.docx]

APPENDIX 1.

***Acutely Presenting Older Patients (APOP)^14^***

1. Age

2. Gender (male/female)

3. Did the patient arrive by ambulance? (yes/no)

4. Needs help with coking/housekeeping? (yes/no)

5. Needs help with bathing? (yes/no)

6. Been admitted to a hospital in the past six months? (yes/no)

7. Diagnosed with dementia? (yes/no)

8. In which year are we living?

9. Say the months in reverse

***Activities of daily living (ADL)^15^***

|  | **Independence**  **(1 point)**  **No supervision, direction or personal assistance.** | **Dependence**  **(0 points)**  **With supervision, direction, personal assistance or total care.** |
| --- | --- | --- |
| **Bathing** | Bathes self completely or needs help in bathing only a single part of the body such as the back, genital area or disabled extremity. | Need help with bathing more than one part of the body, getting in or out of the tub or shower. Requires total bathing. |
| **Dressing** | Get clothes from closets and drawers and puts on clothes and outer garments complete with fasteners. May have help tying shoes. | Needs help with dressing self or needs to be completely dressed. |
| **Toileting** | Goes to toilet, gets on and off, arranges clothes, cleans genital area without help. | Needs help transferring to the toilet, cleaning self or uses bedpan or commode. |
| **Transferring** | Moves in and out of bed or chair unassisted. Mechanical transfer aids are acceptable. | Needs help in moving from bed to chair or requires a complete transfer. |
| **Continence** | Exercises complete self-control over urination and defecation. | Is partially or totally incontinent of bowel or bladder. |
| **Feeding** | Gets food from plate into mouth without help. Preparation of food may be done by another person. | Needs partial or total help with feeding or requires parenteral feeding. |
| **Total points** |  |  |

***Instrumental activities of daily living (IADL)^16^***

|  | **Score** |
| --- | --- |
| **A. Ability to Use Telephone**  1. Operates telephone on own initiative-looks up and dials numbers, etc.  2. Answers telephone but does not dial  3. Does not use telephone at all | 2  1  0 |
| **B. Shopping**  1. Takes care of all shopping needs independently  2. Needs to be accompanied on any shopping trop  3. Completely unable to shop | 2  1  0 |
| **C. Food preparation**  1. Plans, prepares and serves adequate meals independently  2. Needs help with preparation of food  3. Needs to have meals prepares and served | 2  1  0 |
| **D. Housekeeping**  1. Maintains house alone or with occasional assistance (e.g. ‘heavy work domestic help’)  2. Performs light daily tasks such as dish washing, bed making  3. Does not participate in any housekeeping tasks | 2  1  0 |
| **E. Mode of Transportation**  1. Travels independently on public transportation or drives own car  2. Travels on public transportation when accompanied by another  3. Does not travel at all | 2  1  0 |
| **F. Responsibility for own medications**  1. Is responsible for tasking medication in correct dosages at correct time  2. Takes responsibility if medication is prepared in advance in separate dosage  3. Is not capable of dispensing own medication | 2  1  0 |
| **G. Ability to handle finances**  1. Manages financial matters independently (budgets, writes checks, pays rent, bills, goes to bank)  2. Manages day – to- day purchases, but needs help with banking, major purchases, etc  3. Incapable of handling money | 2  1  0 |
| **Total score** |  |

***Clinical Frailty Scale (CFS)^17^***

1. Very Fit – people who are robust, active, energ3etic and motivated. These people commonly exercise regularly. They are among the fittest for their age.

2. Well – people who have no active disease symptoms but are less fit than category 1. Often, they exercise or are very active occasionally, e.g. seasonally.

3. Managing Well – people whose medical problems are well controlled, but are not regularly active beyond routine walking.

4. Vulnerable – while not dependent on others for daily help, often symptoms limit activities. A common complaint is being “slowed up”, and/or being tired during the day.

5. Mildly frail – these people often have more evident slowing, and need help in high order IADLs (finances, transportation, heavy housework, medications). Typically, mild frailty progressively impairs shopping and walking outside alone, meal preparation and housework.

6. Moderately Frail – people need help with all outside activities and with keeping house. Inside, they often have problems with stairs and need help with bathing and might need minimal assistance (cuing, standby) with dressing.

7. Severely frail – completely dependent for personal care, from whatever cause (physical or cognitive). Even so, they seem stable and not a high risk of dying (within ~6 months).

8. Very severely frail – completely dependent, approaching the end of life. Typically, they could not recover even from a minor illness.

9. Terminally ill – approaching the end of life. This category applies to people with a life expectancy < 6 months, who are not otherwise evidently frail.

Scoring frailty in people with dementia

The degree of frailty corresponds to the degree of dementia. Common symptoms in mild dementia include forgetting the details of a recent event, though still remembering the event, itself, repeating the same question/story and social withdrawal.

In moderate dementia, recent memory is very impaired, even though they seemingly can remember their past life events well. They can do personal care with prompting.

In severe dementia, they cannot do personal care without help.

***Caregiver strain index (CSI)^18^***

|  | Yes = 1 | No = 0 |
| --- | --- | --- |
| Sleep is disturbed  (e.g., because… is in and out of bed or wanders around at night) |  |  |
| It is inconvenient  (e.g., because helping takes so much time or it’s a long drive over to help) |  |  |
| It is a physical strain  (e.g., because of lifting in and out of a chair; effort or concentration is required) |  |  |
| It is confining  (e.g., helping restricts free time or cannot go visiting) |  |  |
| There have been family adjustments  (e.g., because helping has disrupted routine; there has been no privacy) |  |  |
| There have been changes in personal plans  (e.g., had to turn down a job; could nog go on vacation) |  |  |
| There have been emotional adjustments  (e.g., because of severe arguments) |  |  |
| Some behaviour is upsetting  (e.g., because of incontinence: … had trouble remembering things; or… accuses people of taking thing) |  |  |
| It is upsetting to find… has changed so much form his/her former self  (e.g., he/she is a different person than he/she used to be) |  |  |
| There have been work adjustments  (e.g., because of having to take time off) |  |  |
| It is a financial strain |  |  |
| Feeling completely overwhelmed  (e.g., because of worry about…; concerns about how you will manage) |  |  |
| **Total Score** |  |  |
